# Breast cancer brain metastasis: Current evidence and future directions

**DOI:** 10.1002/cam4.5021

**Published:** 2022-07-13

**Authors:** Hongna Sun, Junnan Xu, Shuang Dai, Yiwen Ma, Tao Sun

**Affiliations:** ^1^ Department of Medical Oncology, Liaoning Cancer Hospital & Institute Cancer Hospital of China Medical University Shenyang China; ^2^ Department of Medical Oncology, Lung cancer center, West China Hospital Sichuan University Chengdu China

**Keywords:** blood–brain barrier, breast cancer brain metastasis, drug resistance, immunotherapy, molecular mechanisms, targeted therapy

## Abstract

Breast cancer is the most common cancer in women and the second leading cause of cancer‐related deaths after lung cancer. Metastasis of the central nervous system is a terrible event for breast cancer patients, affecting their survival and quality of life. Compared with hormone receptor‐positive/human epidermal growth factor receptor 2‐negative breast cancer patients, brain metastases are more likely to affect patients with triple‐negative breast cancer and human epidermal growth factor receptor 2‐positive breast cancer. The treatment of breast cancer has improved greatly in the last two decades. However, brain metastases from breast cancer remain the leading cause of morbidity and mortality. Patients with breast cancer brain metastasis have been in an inferior position due to the lack of clinical research in this field, and they are often explicitly excluded from almost all clinical trials. The occurrence and progression of brain metastases will result in severe cognitive impairment and adverse physical consequences, so we must have a good understanding of the molecular mechanisms of breast cancer brain metastasis. In this article, we have retrieved the latest literature of molecules and pathways associated with breast cancer brain metastasis, summarized common therapy strategies, and discussed the prospects and clinical implications of targeting the molecules involved.

## Graphical abstract

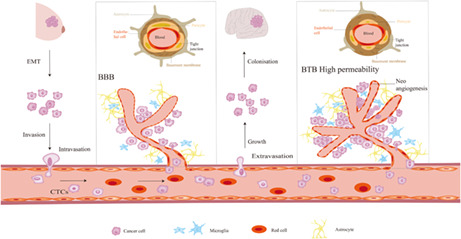

## 1 INTRODUCTION

Breast cancer is the most common cancer in women and the second leading cause of cancer‐related deaths after lung cancer.^1^ With the progress in diagnostic technologies and the advances of molecular‐targeted drugs in clinical practice, the outcomes of metastatic breast cancer have been significantly improved. However, breast cancer brain metastasis (BCBM) is the second most common cause of brain metastasis, and its occurrence has been rising in the past two decades with the significant improvement in survival of advanced breast cancer patients. Brain metastases attack nearly 25% of advanced breast cancer patients, which greatly reduces their quality of life and overall survival (OS).^2^

The risk factors for the development of BCBM are patient characteristics of younger age and ethnicity, tumor features of poorly differentiated, hormone receptor (HR)‐negative and human epidermal growth factor receptor 2 (HER2)‐positive, more than four metastatic lymph nodes and some genetic variations.^3^, ^4^ Breast cancer can spread to bone, liver, lung, and brain, and metastasizing to the brain is a late event. Brain MRI screening is not recommended unless patients have central nervous system (CNS)‐related symptoms of brain metastasis. As a result, detection of brain metastases may be delayed. Therefore, BCBM patients at high risk should be followed up closely. Breast cancer is a heterogeneous disease that can be classified into luminal A, luminal B, HER2‐positive, and triple‐negative subtypes according to receptor status and index of Ki‐67. Each subtype has its own unique growth pattern, natural history, metastatic tendency, and outcome. HER2‐positive and triple‐negative breast cancers (TNBC) are more likely to develop brain metastasis than luminal cancers.^2^ The relationship between different subtypes and BCBM is summarized in Table 1. BCBM patients have been in an inferior position due to the lack of clinical research in this field, and in fact, such patients are often explicitly excluded from almost all clinical trials.

**TABLE 1:** The relationship between various subtypes and BCBM

Subtype	ER	PR	HER2	Ki‐67	Incidence of CNS metastases	mOS after BCBM(months)	Reference
Luminal subtype	(+)	(+)	(−)	Low or high	~15%	7.1 ~ 9.3	2, 45, 120
HER2‐positive	(±)	(±)	(+)	High	~50%	11.5 ~ 18.9	
TNBC	(−)	(−)	(−)	High	~35%	4.4 ~ 4.9	

Abbreviations: BCBM, breast cancer brain metastasis; HER2, human epidermal growth factor receptor 2; ER, estrogen receptor; PR, progesterone receptor; TNBC, triple‐negative breast cancer; +, positive; −, negative; ±, positive, or negative.

Currently, the standard treatment of BCBM is local intervention, including neurosurgical resection and radiation therapy (stereotactic or whole‐brain). While, we use systemic therapies to complement local treatment to better control CNS lesions, and the best management is determined by an experienced multidisciplinary team. However, the outcomes of BCBM patients remain poor because the blood–brain barrier (BBB) limits the penetrability of drugs. It is imperative to detect the underlying molecular mechanisms of BCBM, which will probably provide a basis for preventing or treating such diseases. In this review, we have retrieved the latest literature of molecules and pathways associated with BCBM, summarized common therapy strategies, and discussed the prospects and clinical implications of targeting the molecules involved.

### 1.1 Blood–brain barrier (BBB), blood‐tumor barrier (BTB), and breast cancer metastasize to the brain

The biological structure between blood and brain parenchyma, BBB, separates the blood compartment from brain tissue. Ehrlich et al.^5^ discovered the existence of the BBB for the first time, and subsequent studies provided further details of the structure and function of the BBB. The prominent anatomical architecture of the BBB consists of endothelial cells, pericytes, basement membranes, and astrocytes. The endothelial cells form the blood vessel wall, surrounded intimately by pericytes that are embedded in the basement membrane, and the vessels are ensheathed by astrocytic endfeet.^6^ Besides, the endothelial cells form the tight junctions (TJs) via junctional protein complexes, preventing the paracellular transport,^7^ maintaining CNS homeostasis by tightly allowing the passage of specific nutrients to the brain, restraining the entrance of harmful xenobiotic molecules, and effluxing the toxic substances, metabolites, and waste products.^8^, ^9^ BBB plays a critical role in ensuring normal brain function. BBB is one of the main barriers for cancer cells to extravasate and colonize the brain. However, as the development of primary or metastatic tumors in the brain, relevant changes occur in this context: new aberrant vessels grow during tumor progression, and the BBB becomes disrupted and is altered to the BTB.^10^ We know little about BTB, and most of our understanding of the microenvironment of CNS neoplasms originates from rodent models. BTB is highly heterogeneous and easier to leak than the BBB, which is the basis for drugs entering the brain. Anatomically, BTB is featured by abnormal pericyte distribution, alteration of the basement membrane, loss of astrocytic endfeet, and neuronal connections. Functionally, BTB is characterized by non‐uniform permeability, which results from uneven distribution of drugs in mouse models of CNS metastasis.^11^ BTB is not an autonomous structure because it occurs synchronously with cancer cells and is affected by cancer cell behaviors.

**FIGURE 1:**
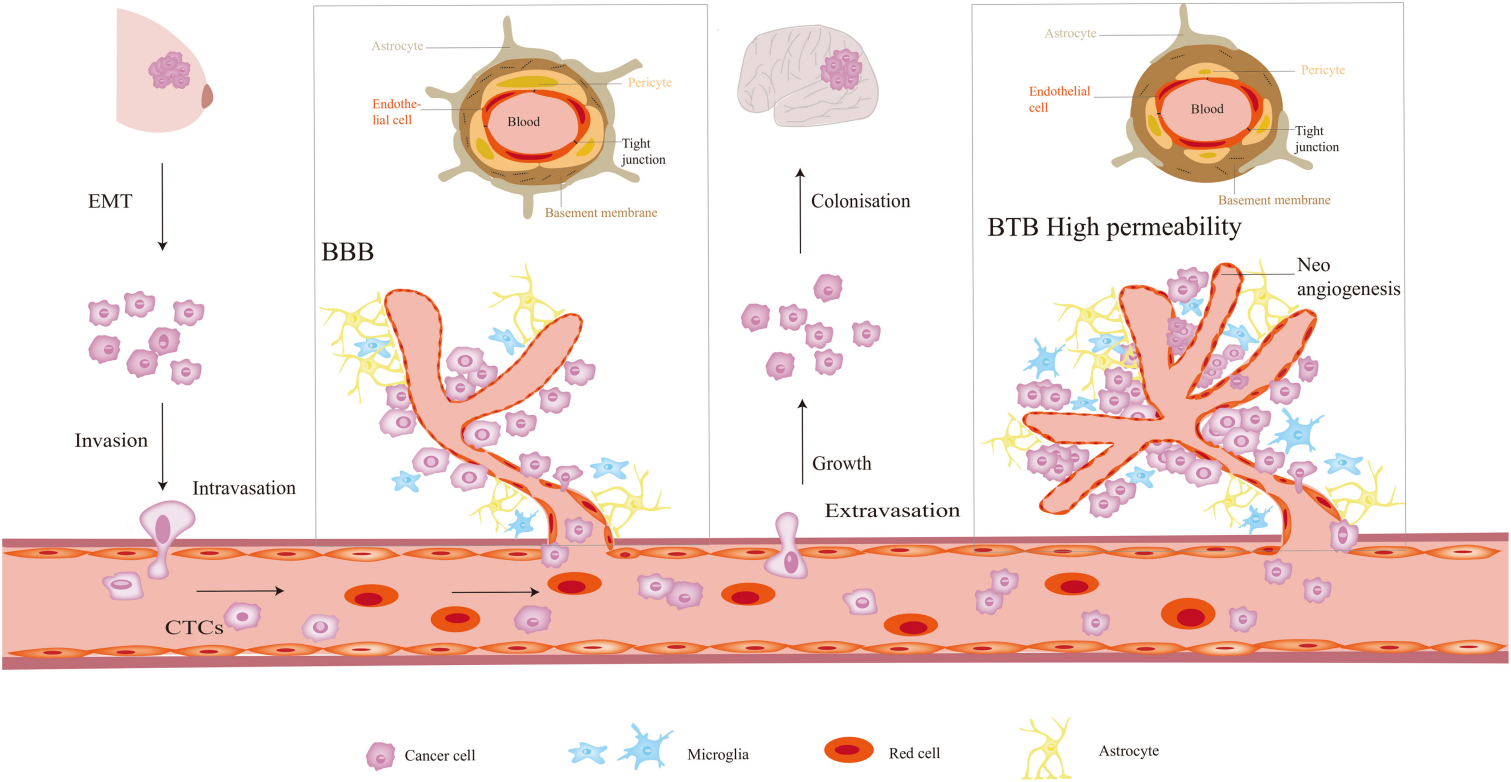
Breast cancer cell metastasis to the brain. A portion of cells at the primary site acquired invasive properties by EMT. Invasive cancer cells intravasate into the bloodstream, survive, and arrest the circulatory system. Then these cells extravasate through transendothelial migration, colonize, and form metastatic brain lesions. BBB plays a critical role in ensuring normal brain function. However, as the development of primary or metastatic tumors in the brain, BBB becomes disrupted, and is altered to BTB. At last, new, and aberrant vessels grow during tumor progression. Abbreviations: EMT, epithelial to mesenchymal transition; BBB, blood‐brain barrier; BTB, blood tumor barrier.

The development of brain metastasis is caused by a series of complicated and multistage orchestrated cellular processes (Figure 1). At first, morphology and adhesion of cells are changed by epithelial‐mesenchymal transition (EMT), which is an essential step to start metastasis. Breast cancer cells acquire traits of mesenchymal cells, allowing invasion, intravasation, and distant metastasis.^12^ Therefore, the tumor cells are more likely to escape from the primary tumor. Then, the tumor cells invade from the basement membrane to surrounding tissues, intravasate into the bloodstream or lymphatic vessels, survive, and arrest in the circulatory system, extravasate through transendothelial migration, colonize, and eventually form distant metastatic lesions.^13^

## 2 GENE MUTATIONS AND SIGNALING PATHWAYS INVOLVED IN BCBM

Various aberrant genes and signaling pathways are involved in metastatic breast cancer, which possibly acts as promising biomarkers to predict relapse and provide a targeted therapy strategy. However, the molecular mechanism of breast cancer metastasis, especially brain metastasis, has not been fully clarified yet. To better understand these diseases, we have reviewed related literature and summarized the signaling pathways and mechanisms associated with the development of BCBM, hoping to provide new perspectives for targeted therapy of BCBM.

### 2.1 Wnt and notch signaling pathway

Wnt and Notch pathways play a protective role in normal stem cells and are also connected with tumor stem cells. There are three different activation pathways of Wnt signaling: beta‐Catenin‐dependent pathways (canonical WNT pathway), planar cell polarity (PCP) pathways, and Wnt/Ca2+ pathways. Activation of non‐typical WNT signaling is related to the invasive behavior of the basal‐like subtype.^14^, ^15^ Smid et al. found that members of the Wnt signaling are highly expressed in basal‐like breast cancer and brain‐specific relapse, suggesting that the active Wnt/β‐catenin pathway may be helpful to basal‐like breast cancer metastasis to the brain.^15^ Klemm et al. also discovered upregulated Wnt pathways were closely correlated to basal‐like and other subtypes of breast cancers metastasis to CNS.^14^ Multiple studies have shown that Notch signaling pathways act in either an oncogenic or a tumor‐suppressive manner in cancer cells. Classical NOTCH pathways are composed of four NOTCH receptors (NOTCH1‐4) and corresponding ligands (Delta‐like 1, 3, and 4 and Jagged 1 and 2). Nam et al. cultured a brain metastasis model of breast cancer using the breast cancer cell line MDA‐MB‐435. They discovered that high expression of the Jagged‐2 ligand could activate the Notch pathway in Br4, which promoted tumor cell migration and invasion, suggesting that the activation of the Notch pathway might play an essential role in CNS metastasis.^16^ Also, Xing et al. found that IL‐1β was highly expressed in metastatic brain cells, which was associated with tumor angiogenesis, growth, and invasion. IL‐1β played a key role in metastasis by upregulating the expression of Notch ligand JAG1 in astrocytes. The interaction of astrocytes and cancer stem‐like cells significantly inhibited Notch signaling in cancer stem‐like cells. Furthermore, they found that compound E, a BBB permeable Notch inhibitor, could substantially inhibit brain metastasis. The discovery provided an opportunity to identify a novel therapeutic target for BCBM.^17^ In addition, Leontovich et al. demonstrated that NOTCH3 could enhance the invasive ability of unique TNBC cells (TNBC‐M25) originated from a patient‐derived CNS metastasis.^18^ Increasing evidences have indicated that Wnt and Notch signaling are of great significance in the regulation of BCBM, while there is little clinical experience about Wnt and Notch pathway inhibitors.

### 2.2 PI3K/AKT/mTOR signaling pathway and PTEN

The phosphatidylinositol‐3 kinase (PI3K)/AKT/mammalian target of rapamycin (mTOR) signaling pathway affects such biological functions as cell proliferation, growth, metabolism, angiogenesis, invasion, migration, and apoptosis. Phosphatidylinositol‐4,5‐bisphosphate 3‐kinase catalytic subunit alpha (PIK3CA) has been confirmed as a category of oncogenes, which encode the catalytic subunit P110 of PI3K. When the PIK3CA gene occurs mutation, loss, or amplification, the abnormal P110 subunit will be encoded, resulting in the continuous activation of PI3K.^19^ PI3K is a member of the lipid kinases family. PI3K can be divided into three categories based on structural features and lipid substrate preferences. Class I PI3Ks include four isoforms: p110α, p110β, p110γ, and p110δ, which are also known as PIK3CA, PIK3CB, PIK3CG, and PIK3CD, respectively. Class I PI3Ks appear in all cell types, while δ and γ are highly enriched in leukocytes.^20^ Class II PI3Ks have three isoforms: PI3K‐C2α, PI3K‐C2β, and PI3K‐C2γ. α and β are expressed in most of the tissues, however, a research reported that γ is preferentially expressed in the liver.^21^ Class III PI3Ks has one member: VPS34. Among them, the class I PI3K, especially PIK3CA, is concerned with the development of breast cancer. About 30–40% of breast cancer patients possess PIK3CA mutations, and hotspot mutations are mainly located in exons 9 and 20.^22^ AKT, the serine/threonine‐protein kinase, is the primary downstream molecule of the PI3K pathway. AKT is activated by PtdIns(3,4)P2 (PIP2) and PtdIns(3,4,5)P3 (PIP3) directly binding to the pleckstrin homology domain of AKT. After activation, AKT further phosphorylates its downstream substrate, regulating cell proliferation, invasion, apoptosis, and glycogen metabolism.^22^ mTOR, a class of serine/threonine kinases, has been identified as the downstream target of PI3K/AKT, and it acts on a variety of signaling pathways by regulating transcription and albumin synthesis.^19^ There is a complex of mTORC1 and mTORC2 in the cell. mTORC1 promotes cell growth and the progression of cell cycle. mTORC2 regulates cell survival, metabolism, and cytoskeleton construction.^23^

PI3K/AKT/mTOR axis influences cell growth, survival, motility, and metabolism of breast cancer. Furthermore, PI3K/AKT/mTOR signaling pathway plays a significant role in regulating CNS metastasis.^24^ A systematic review comprehensively reported the most frequently mutated genes discovered in samples of BCBM and found that PIK3CA (22%) was the second most commonly reported gene, after TP53 (52%).^25^ PI3K/AKT/mTOR pathway can be activated in metastatic cells as well as in the metastatic microenvironment. Microglia expresses Class I PI3Ks, forming a heterodimer, which includes a catalytic subunit (p110) and a regulatory subunit (p85). The regulatory subunit binds to the relevant receptors and the catalytic subunit phosphorylates PIP2 to PIP3 and then activates the downstream pathway AKT, which inhibits apoptosis and contributes to cell survival. A study found that PI3K/AKT/mTOR pathway resulted in the overexpression of immunorelated genes (*PD‐L1*, *CSF1*, and *CSF1R*) or cytotoxic T lymphocyte‐associated protein 4 (CTLA4) in microglia or cancer cells in the microenvironment of brain metastases. The expression of these genes and the invasive cancer cells of BCBM are significantly decreased when using a pharmacological inhibitor of the PI3K/AKT/mTOR signaling pathway.^26^

Phosphatase and tensin homolog (PTEN), a lipid phosphatase that eliminates the 3‐phosphate from PIP2 and PIP3, negatively regulates the PI3K/AKT pathway. Consequently, the deletion of PTEN can activate the PI3K/AKT signaling cascade by restraining the degradation of PIP2 and PIP3. Compared with HR+/HER2‐ and HER2+ breast cancer, loss of PTEN is more common in TNBC.^27^, ^28^ The deletion of PTEN may lead to the dismal OS in TNBC patients with BCBM.^29^ Wikman et al. demonstrated that the expression of PTEN was significantly decreased in CNS metastases compared to nonmetastatic primary tumors. Moreover, the frequency of the mutation analysis of the PTEN gene in BCBM was much higher than that in primary tumors.^30^ Zhang et al. discovered that primary tumor cells expressing normal levels of PTEN would lose PTEN expression after spreading to the brain, not to other organs. In addition, after leaving the brain microenvironment, the expression level of PTEN in PTEN‐loss brain metastatic cancer cells was restored. Moreover, they also found that this process was regulated by microRNAs (miRNAs) from astrocytes. Furthermore, loss of PTEN in brain metastatic tumor cells increased the expression of cytokine chemokine (C‐C motif) ligand 2 (CCL2), which promoted the development of brain metastatic tumor cells.^31^

### 2.3 ERBB signaling pathway

EGFR (epidermal growth factor receptor, also known as ERBB1/HER1), ERBB2 (HER2), ERBB3 (HER3), and ERBB4 (HER4) are members of the ERBB family of receptor tyrosine kinase (RTKs). These four receptors are similar in structure, comprising a transmembrane segment, an intracellular protein tyrosine kinase domain, and an extracellular ligand‐binding domain.^32^ ERBB family members participate in regulating vital biological processes, including cell differentiation, proliferation, angiogenesis, migration, survival, apoptosis, and metabolism through activating downstream signaling pathways, such as PI3K/Akt, Ras/MEK/ERK, Janus‐activated kinase/signal transducer, activator of transcription (JAK/STAT), and phospholipase Cγ (PLCγ)/PKC.^33^ Among the members of the ERBB family, HER2, and EGFR are often highly expressed in multiple cancers. Activation of HER2‐mediated signaling pathways is induced by heterodimers of HER2‐EGFR or HER2‐HER3, or by HER2 homodimers instead of straightly binding to any known ligands, which is different from other ERBB family members.^34^ Furthermore, HER3 is a critical partner for HER2‐amplified breast cancer tissues. An ERBB signaling pathway is activated by various mechanisms, including constitutive activation of receptors, excess of receptors, and excess of ligands.^33^ The signal transfer process can be summarized as follows: ligand binding to the extracellular domain and exposing the dimerization domain to allow receptor dimerization. Then each receptor activates its partner through phosphorylation, accompanied by tyrosine kinase section of the dimer moiety transactivation. At the end, the phosphorylation event activates downstream signaling pathways.^34^

EGFR, HER2, and ERBB3 are all related to the causation and progression of cancer. However, the role of ERBB4 in oncogenesis remains less well defined. Among the four receptors, ERBB4 is unique as it is the only member with a growth inhibition effect. EGFR mutations (L858R point mutation and exon 19 deletion) in lung cancer have been well studied and testified to have good effects of EGFR tyrosine kinase inhibitor (TKI) such as gefitinib and erlotinib. Compared with lung cancer, EGFR mutation hardly happens in breast cancer.^35^ Hohensee et al. displayed that EGFR mutations were relatively more common in brain metastasis than other distant metastases or primary tumors, suggesting that TNBC patients with EGFR variation are at high risk of developing brain metastases.^28^ Researches concerning ERBB3 are far less than HER2. However, the number of ERBB3 studies is gradually increasing and substantial studies are concentrated on developing new therapies that target ERBB3. ERBB3 is regularly expressed in human breast cancers accompanied by HER2.^34^ P. Kodack et al. demonstrated that the resistance to PI3K inhibitor would take place in PI3KCA‐mutations and/or HER2‐amplification BCBM when the activity of the ERBB3 signaling pathway was enhanced in vivo or in vitro. Blocking ERBB3 decreased the activity of PI3K and the relevant downstream pathway, and recovered the efficacy of the PI3K inhibitor, implying that the activation of the PI3K‐AKT pathway by ERBB3 could lead to CNS metastasis.^36^ U3–1402 (patritumab deruxtecan), a novel ERBB3 inhibitor, is an ERBB3‐targeted antibody‐drug conjugate consisting of a novel topoisomerase I inhibitor, DX‐8951 derivative (DXd), and the HER3 antibody patritumab. U3–1402 exhibits antitumor activity in several cancers.^37^, ^38^, ^39^, ^40^ So far, patritumab deruxtecan has been explored in advanced breast cancer patients with HER3 overexpression. In the 2022 ASCO annual meeting, investigators reported the updated safety and efficacy data from the phase 1/2 study of patritumab deruxtecan in patients with HER3‐expressing metastatic breast cancer. Although this population had been highly pretreated, patritumab deruxtecan demonstrated promising activity in patients with advanced HR+/HER2−, HER2+, and TNBC patients. Furthermore, the longer follow‐up safety profile revealed adequate safety and tolerability.^41^

HER2 overexpression is mainly attributed to HER2 gene amplification and constitutive activation of the HER2 signaling network.^33^ Apart from gene amplification, HER2 overexpression can be influenced by other potential mechanisms. For instance, FOXP3, an X‐linked tumor suppressor gene, plays an essential role in keeping low levels of HER2. Therefore, the mutation or absence of FOXP3 promotes overexpression of HER2.^42^ Previous studies have confirmed that HER2‐positive is an important prognostic and predictive factor in the development of BCBM. HER2 overexpression can be seen in about 30% of breast cancer patients and is related to the advanced condition and poor OS.^43^ Furthermore, brain metastases will happen to approximately 50% of patients with HER2+ breast cancer, with a median survival of 7 to 18 months after diagnosis.^44^, ^45^, ^46^ There are three factors to explain the propensity of metastasis to CNS in HER2‐positive breast cancer patients. First, anti‐HER2 therapy extends the survival of patients, which in turn brings about brain metastases. Second, the limited permeability of the BBB by trastuzumab makes the brain a “sanctuary” site for metastases. Third, HER2‐positive breast cancer has the inherent tendency of metastasis to the brain. Palmieri et al. put forward that HER2 overexpression would have impact on the natural history of breast cancer brain metastatic growth by transfecting HER‐2 into 231‐ BR cells (a brain‐seeking breast cancer cell line), which significantly increased brain metastatic colonization.^47^

## 3 POTENTIAL STRATEGIES FOR PREVENTION OR TREATMENT OF BCBM

Patients are prone to develop CNS metastasis, even though their extracranial lesions are controlled. Approximately half of the patients succumb to brain metastasis. Unfortunately, BCBM patients are routinely excluded from clinical trials. There are few targeted treatment options for BCBM. Nevertheless, with the wide application of second‐generation sequencing, underlying genetic mutations have been discovered in clinical practice. What's more, the strategies for prevention and treatment of BCBM have been further developed along with a better understanding of the BBB and the application of targeted drugs such as monoclonal antibodies, tyrosine kinase inhibitors, PARP inhibitors, immune checkpoint inhibitors, and CDK4/6 inhibitors. Several clinical trials are ongoing to investigate new drugs or combinations treating BCBM. We select some ongoing clinical trials in Table 2.

**TABLE 2:** Selected ongoing clinical trials of targeted therapy and immunotherapy in patients with BCBM

Treatment	NCT identifier	Title	Phase	Number of patients	Primary point
HER2 targeted drug	NCT02614794	Phase 2 Randomized, Double‐Blinded, Controlled Study of Tucatinib vs Placebo in Combination With Capecitabine and Trastuzumab in Patients With Pretreated Unresectable Locally Advanced or Metastatic HER2+ Breast Carcinoma	II	612	PFS
	NCT03975647	Randomized, Double‐blind, Phase 3 Study of Tucatinib or Placebo in Combination With Ado‐trastuzumab Emtansine (T‐DM1) for Subjects With Unresectable Locally advanced or Metastatic HER2+ Breast Cancer (HER2CLIMB‐02)	III	460	PFS
	NCT03933982	Pyrotinib Plus Vinorelbine in Patients With Brain Metastases From HER2‐positive Metastatic Breast Cancer: a Prospective, Single‐arm, Open‐label Study	II	30	CNS ORR
	NCT03691051	Pyrotinib Plus Capecitabine in Patients With Brain Metastases From HER2‐positive Metastatic Breast Cancer: a Single‐arm, Open‐label, Ahead Study	II	78	CNS ORR
	NCT04303988	A Prospective, Single‐arm, Single‐center, Multi‐cohort Phase II Clinical Study of HER2‐positive and Triple‐negative BCBM	II	59	CNS ORR
	NCT01494662	A Phase II Trial of HKI‐272 (Neratinib), Neratinib, and Capecitabine, and Ado‐Trastuzumab Emtansine for Patients With Human Epidermal Growth Factor Receptor 2 (HER2)‐Positive Breast Cancer and Brain Metastases	II	168	ORR
	NCT02536339	An Open‐Label, Single‐Arm, Phase II Study of Pertuzumab With High‐Dose Trastuzumab for the Treatment of Central Nervous System Progression Post‐Radiotherapy in Patients With HER2‐Positive Metastatic Breast Cancer (PATRICIA)	II	40	CNS ORR
	NCT03417544	A Phase II Study of Atezolizumab in Combination With Pertuzumab Plus High‐dose Trastuzumab for the Treatment of Central Nervous System Metastases in Patients With Her2‐positive Breast Cancer	II	33	CNS ORR
	NCT01622868	Phase II Randomized Study of Whole Brain Radiotherapy/Stereotactic Radiosurgery in Combination With Concurrent Lapatinib in Patients With Brain Metastasis From HER2‐Positive Breast Cancer ‐ A Collaborative Study of NRG Oncology and KROG	II	143	CR Rate in the Brain at 12 Weeks after RT
	NCT03190967	Phase I/II Study of T‐DM1 Alone Versus T‐DM1 and Metronomic Temozolomide in Secondary Prevention of HER2‐Positive Breast Cancer Brain Metastases Following Stereotactic Radiosurgery	I/II	125	Maximum tolerated dose of TMZ; Median time to progression
	NCT03054363	Phase IB/II Open‐label Single‐Arm Study to Evaluate Safety and Efficacy of Tucatinib in Combination With Palbociclib and Letrozole in Subjects With Hormone Receptor‐Positive and HER2‐positive Metastatic Breast Cancer	I/II	42	Phase I: safety and tolerability Phase II: PFS
PI3K inhibitor	NCT03765983	Phase II Trial of GDC‐0084 in Combination With Trastuzumab for Patients With HER2‐Positive BCBM	II	47	CNS ORR
	NCT02000882	Phase II Multicenter Single‐arm Study of BKM120 Plus Capecitabine for Breast Cancer Patients With Brain Metastases	II	10	CBR
mTOR inhibitor	NCT01305941	A Phase II Study Evaluating The Efficacy And Tolerability Of Everolimus (RAD001) In Combination With Trastuzumab And Vinorelbine In The Treatment Of Progressive HER2‐Positive BCBM	II	32	CNS ORR
	NCT01283789	Phase II Trial of Lapatinib and RAD‐001 for HER2 Positive Metastatic Breast Cancer	II	23	Efficacy
	NCT01783756	Phase 1b/2 Single‐arm Trial Evaluating the Combination of Lapatinib, Everolimus, and Capecitabine for the Treatment of Patients With HER2‐positive Metastatic Breast Cancer With CNS Progression After Trastuzumab	I/II	9	CNS ORR
CDK4/6 inhibitor	NCT02896335	A Phase 2 Study of Palbociclib in Progressive Brain Metastases Harboring Alterations in the CDK Pathway	II	30	CBR
	NCT02308020	A Phase 2 Study of Abemaciclib in Patients With Brain Metastases Secondary to Hormone Receptor‐Positive Breast Cancer, Non‐small Cell Lung Cancer, or Melanoma	II	162	CNS ORR
	NCT04334330	Palbociclib, Trastuzumab, Lapatinib and Fulvestrant Treatment in Patients With Brain Metastasis From ER Positive, HER‐2 Positive Breast Cancer: A Multi‐center, Prospective Study in China	II	48	CNS ORR
PARP inhibitor	NCT02595905	Phase II Randomized Placebo‐Controlled Trial of Cisplatin With or Without ABT‐888 (Veliparib) in Metastatic Triple‐Negative Breast Cancer and/or BRCA Mutation‐Associated Breast Cancer, With, or Without Brain Metastases	II	333	PFS
	NCT01173497	A Phase II Study of the PARP Inhibitor, INIPARIB (BSI‐201), in Combination With Chemotherapy to Treat Triple‐Negative BCBM	II	44	Efficacy
	NCT04508803	Combination of HX008 And Niraparib in germ‐line‐mutated metastatic breast cancer: a multi‐center Phase II study	II	50	ORR
Immunotherapy	NCT04303988	A Prospective, Single‐arm, Single‐center, Multi‐cohort Phase II Clinical Study of HER2‐positive and Triple‐negative BCBM	II	59	CNS ORR
	NCT03449238	Pembrolizumab And SRS Of Selected Brain Metastases In Breast Cancer Patients	I/II	41	Tumor response for non‐irradiated brain lesions at 8 weeks
	NCT03483012	A Phase II Study of Atezolizumab in Combination With SRS for Patients With Triple‐negative Breast Cancer and Brain Metastasis	II	45	PFS
Other therapies	NCT03696030	A Phase 1 Cellular Immunotherapy Study of Intraventricularly Administered Autologous HER2‐Targeted Chimeric Antigen Receptor (HER2‐CAR) T Cells in Patients With Brain and/or Leptomeningeal Metastases From HER2 Positive Cancers	I	39	Incidence of dose‐limiting toxicities; treatment‐related adverse events
	NCT04158947	A Randomized Study of HER2+ Breast Cancer Patients With Active Refractory Brain Metastases Treated With Afatinib in Combination With T‐DM1 vs. T‐DM1 Alone	I/II	130	Safety and tolerability
	NCT03613181	A Randomized Open‐Label, Multi‐Center Pivotal Study of ANG1005 Compared With Physician's Best Choice in HER2‐Negative Breast Cancer Patients With Newly Diagnosed Leptomeningeal Carcinomatosis and Previously Treated Brain Metastases (ANGLeD)	III	150	OS
	NCT02581839	Treatment of Brain Metastases From Breast Cancer With Eribulin Mesylate	II	9	CNS PFS
	NCT02260531	A Phase II Study of Cabozantinib Alone or in Combination With Trastuzumab in Breast Cancer Patients With Brain Metastases	II	36	CNS ORR

Abbreviation: BCBM, breast cancer brain metastases; CBR, clinical benefit rate; CNS, central nervous system; ORR, objective response rate; OS, overall survival; PFS, progression‐free survival; RT, radiation therapy; SRS, stereotactic radiosurgery; TMZ, temozolomide; T‐DM1, trastuzumab Emtansine.

### 3.1 PI3K/AKT/mTOR signaling pathway

A few studies have addressed that PI3K/AKT/mTOR pathway occurs in 43–75% of BCBM patients, indicating that inhibiting this pathway may be a helpful treatment strategy for BCBM patients.^29^, ^48^, ^49^

Buparlisib, a potent pan‐class I PI3K inhibitor, has demonstrated its effectiveness in postmenopausal patients with HR+ breast cancer refractory to aromatase inhibitors.^50^, ^51^ Like capecitabine, buparlisib can also cross the BBB, making it a preferred candidate for treating BCBM patients. A phase II clinical trial was made to evaluate the safety and effectiveness of buparlisib plus capecitabine in BCBM, which is ongoing (NCT02000882). Maria Ippen et al. have proven that GDC‐0068, an ATP‐competitive pan‐AKT inhibitor, induces apoptosis and presents a robust tumor‐suppressing role in PIK3CA‐mutant BCBM xenograft models, which provides a significant survival benefit, implying that GDC‐0068 may be a promising targeted therapy strategy for BCBM patients with mutations in the PI3K pathway.^52^ Everolimus, a brain‐permeable mTORC1 inhibitor, has been approved in combination with exemestane for patients previously treated with nonsteroidal aromatase inhibitors and HR+/HER2‐ advanced breast cancer. Everolimus is effective in HR+/HER2‐ terminal breast cancer in BOLERO‐2^53^ and BOLERO‐3^54^ trials. However, both trials excluded BCBM patients. The role of everolimus in BCBM has been researched in several studies.^55^, ^56^ Phase Ib/II trial (TRIO‐US B‐09) revealed that the combination of lapatinib, everolimus, and capecitabine was efficient in refractory HER2+ BCBM with a CNS objective response rate (ORR) of 27% at 12 weeks and a progression‐free survival (PFS) of 6.2 months.^55^ A trial of studying the combination of everolimus, vinorelbine, and trastuzumab in heavily pretreated patients population of HER2+ BCBM showed limited activity in the intracranial lesions.^56^ In short, further researches on targeting the PI3K/AKT/mTOR signaling for BCBM patients remain needed.

### 3.2 HER2 signaling pathway

Before the era of HER2‐targeted therapy, HER2+ breast cancer was invasive with rapid recurrence and poor survival. The application of anti‐HER2 targeted drugs has dramatically increased the survival of this subtype. Anti‐HER2 drugs can be classified into monoclonal antibodies (trastuzumab and pertuzumab), antibody‐drug conjugates (T‐DM1 and T‐DXd), and small‐molecule tyrosine kinase inhibitors (tucatinib, lapatinib, neratinib, and pyrotinib). These are widely applied in clinical practice. Table 3 provides the most pivotal clinical trials of patients with HER2+ BCBM.

**TABLE 3:** Important clinical trials in patients of HER2‐positive BCBM

Clinical trials	Agents	Trial characteristics	Number of patients	CNS ORR	mPFS	mOS	Time to BM after medication (m)	Incidence of brain metastases
Krop et al.^64^ Phase III NCT00829166	Trastuzumab Emtansine (T‐DM1) vs. lapatinib+capecitabine (XL)	An exploratory analysis of patients with CNS metastases in EMILIA	T‐DM1: 45 XL: 50	NR NR	5.9 5.7	26.8 12.9	NR NR	NR NR
Montemurro et al.^65^ Phase IIIb NCT01702571	Trastuzumab Emtansine (T‐DM1)	A post hoc exploratory analysis of KAMILLA trial	126	21.4%	5.5	18.9	NR	NR
Swain et al.^62^ Phase III NCT00567190	Pertuzumab+trastuzumab+ docetaxel (PTD) vs. placebo+trastuzumab+docetaxel (TD)	An exploratory analysis of CLEOPATRA trial	PTD: 51 TD: 55	NR NR	NR NR	34.4 26.3	15.0 11.9	13.7% 12.6%
Bachelot et al.^82^ Phase II NCT00967031	Lapatinib+capecitabine	LANDSCAPE: study for newly diagnosed brain metastases	45	65.9%	5.5	17	NR	NR
Freedman et al.^83^ Phase II NCT01494662	Neratinib+capecitabine	TBCRC 022: study for HER2+ breast cancer and brain metastases	pretreated lapatinib? NO:37 YES:12	49% 33%	5.5 3.1	13.3 15.1	NR NR	NR NR
Hurvitz et al.^85^ Phase III NCT01808573	neratinib+capecitabine (NC) vs. lapatinib+capecitabine (LC)	Efficacy of NC in the subgroup of patients with BM from NALA Trial	NC: 51 LC: 50	26.0% 15.0%	7.8 5.5	48.0 16.4	26.2% 41.6%	NR NR
Yan et al.^88^ Phase II NCT03691051	Pyrotinib+capecitabine	PERMEATE: study for newly diagnosed brain metastases	previous radiotherapy? NO: 59 YES: 19	74·6% 42.1%	11.3 5.6	NR NR	NR NR	NR NR
Murthy et al.^76^ Phase II NCT02614794	Tucatinib+trastuzumab+capecitabine (TTC) vs. placebo+trastuzumab+capecitabine (TC)	HER2CLIMB:Tucatinib+trastuzumab+capecitabine for HER2+ breast cancer	TTC:198 TC:93	47.3% 20.0%	9.9 4.2	18.1 12.0	NR NR	NR NR
Jerusalem et al.^67^ Phase II NCT03248492	T‐DXd	A subgroup analysis of the DESTINY‐Breast01 trial	24	58.3%	18.1	NR	NR	NR

Abbreviations: BM, brain metastasis; CNS, central nervous system; HER2, human epidermal growth factor receptor; m, months; mPFS, median progression‐free survival; mOS, median overall survival; NR: not reported; ORR, objective response rate; ph, phase; T‐DXd, trastuzumab deruxtecan; vs., versus.

Trastuzumab, a recombinant humanized monoclonal antibody blocking HER2 receptors, can recognize and bind to the extracellular domain of HER2 receptors, thus weakening the proliferation of tumor cells. Trastuzumab is the first approved targeted drug for treating HER2+ breast cancer in clinical practice and is now widely used as the first‐line therapy.^57^ The application of trastuzumab has dramatically changed the natural history of HER2+ breast cancer. Multiple studies have shown that trastuzumab can significantly prolong the time to develop CNS metastasis of HER2+ breast cancer.^58^, ^59^ However, a meta‐analysis of four randomized trials among 9020 patients has revealed that adjuvant trastuzumab may increase the risk of CNS metastases as the first relapse location in HER2+ breast cancer patients,^60^ because the drug cannot cross the BBB. Alternatively, it might lead to more brain metastasis as trastuzumab enhances extracranial lesions control and prolongs survival.

Pertuzumab, another humanized monoclonal antibody targeting HER2, plus trastuzumab and docetaxel has been widely used in terminal breast cancer as first‐line therapy.^61^ An exploratory analysis of the CLEOPATRA study indicates that trastuzumab, pertuzumab, and docetaxel cannot decrease the incidence of brain metastases but can delay the development of brain metastasis compared with trastuzumab, docetaxel, and placebo (15 vs. 11.9 m).^62^ However, after radiotherapy, dual‐target trastuzumab and pertuzumab produces disappointing outcomes against brain metastases for the difficulties in CNS penetration of monoclonal antibodies.^63^ Trastuzumab emtansine (T‐DM1), an antibody drug conjugate consisting of trastuzumab and cytotoxic agent DM1, is approved as second‐line therapy for patients pretreated by trastuzumab, pertuzumab, and taxane. A retrospective exploratory analysis of EMILIA suggested that the PFS of brain metastasis in patients with HER2+ terminal breast cancer was similar to that of T‐DM1 and lapatinib–capecitabine (5.9 vs. 5.7 m).^64^ Similarly, A post hoc exploratory analysis of KAMILLA showed that median PFS was 5.5 months in HER2+ BCBM patients treated with TDM1.^65^ TDM1 seems to be active in brain lesions in spite of lower OS than the patients without intracranial diseases.

Trastuzumab deruxtecan (T‐DXd, DS8201) is an antibody‐drug conjugate composed of a topoisomerase I inhibitor and an anti‐HER2 antibody. The DESTINY‐Breast01 trial demonstrated that T‐DXd had strong anti‐tumor activity in pretreated patients with HER2+ metastatic breast cancer.^66^ And DESTINY‐Breast01 subgroup analysis revealed a median PFS of 18.1 months in HER2+ BCBM patients treated with T‐DXd, which suggested that it would be a promising therapeutic strategy.^67^ The latest data of DESTINY‐Breast03 showed that T‐DXd was superior to TDM1 in patients with HER2+ advanced breast cancer who had been previously treated with trastuzumab and a taxane. And the subgroup analysis also suggested that patients with brain metastasis had a significant benefit from T‐DXd.^68^ In addition, despite a more extended treatment duration with T‐DXd, it demonstrated a tolerable safety profile in the result of safety follow‐up of the study of DESTINY‐Breast03, which was reported in the 2022 ASCO meeting abstract.^69^ Although T‐DXd's intracranial response and long‐term clinical activity in HER2+ metastatic breast cancer patients were emphasized in DESTINY‐Breast01 and DESTINY‐Breast03, both studies did not include patients with active BCBMs. In the latest reported results of the DEBBRAH trial, T‐DXd presented the intracranial activity of HER2+ metastatic breast cancer patients with active and asymptomatic brain metastasis with an intracranial ORR of 44.4% and 50.0%, respectively.^70^ In addition, the TUXEDO‐1 trial studied T‐DXd in HER2+ breast cancer patients with active brain metastasis, showing that intracranial RR was 73.3% (11/15) and PFS was 14 months (95%CI 8.48–19.52) at 11 months median follow‐up. The results suggest that T‐DXd achieves significant therapeutic effects in CNS metastasis and should thus be further explored in this context.^71^ DESTINY‐Breast04 demonstrated a statistically significant and clinically meaningful benefit of T‐DXd in PFS and OS compared to standard‐of‐care treatment in patients with HER2‐low metastatic breast cancer.^72^ Therefore, knowing the proportion of low expression of HER2 in BCBM is significant for determining the targeted therapy in such patients.

CNS metastases occur in approximately 35–62% of patients with HER2+ advanced breast cancer after being treated with trastuzumab, thereby resulting in a poor prognosis.^58^, ^73^, ^74^ In addition, studies of patients carrying intracranial lesions were conducted to compare the concentration of trastuzumab in cerebrospinal fluid (CSF) and plasma, which showed that trastuzumab levels in their plasma were much higher than those in cerebrospinal fluid.^75^ To sum up, these data may explain why brain metastasis still occurs when trastuzumab effectively controls the extracranial disease. An intact BBB may hinder the penetration of macromolecule drugs like trastuzumab into the brain. Besides antibody drugs, a growing number of small‐molecule TKIs have been testified efficiency or are under evaluation at different phases of pre‐clinical and clinical studies. Compared to antibody drugs, TKIs are easier to penetrate to CNS due to a small molecular weight, thus producing an antitumor effect on the brain.

Tucatinib, an oral KTI, has been demonstrated with high efficiency in BCBM patients. In the HER2CLIMB clinical trial, researchers applied tucatinib plus trastuzumab and capecitabine to heavily pretreated patients with HER2+ advanced breast cancer, including a large percentage of patients with brain metastases, which obtained significant clinical benefit. The result implies that compared with targeting the external domain alone, targeting the internal domain of HER2 with tucatinib and the external domain with trastuzumab at the same time remarkably improves the survival of patients.^76^ In addition, tucatinib plus trastuzumab and capecitabine was the first drug approved by Food and Drug Administration (FDA) to treat BCBM patients. Considering the significant therapeutic effect of tucatinib on the CNS in the HER2CLIMB trial, the COMPASS‐RD trial is ongoing to test the combined application of T‐DM1 and tucatinib in the high‐risk residual lesion setting (NCT03975647), hoping that it will enhance disease‐free survival and control CNS progression. In addition, a recent study by Cordero et al. proved that tucatinib plus LM008–HER2Ab neural stem cells could continuously secrete abundant anti‐HER2Ab, and through inhibiting PI3K/Akt signaling, significant survival benefit was achieved in the preclinical models of HER2+ BCBM.^77^

Lapatinib is a small dual TKI of HER1 and HER2. Lapatinib plus capecitabine is approved to treat metastatic HER2‐positive breast cancer that progresses after trastuzumab treatment. Morikawa found that lapatinib could cross the BBB for the first time.^78^ However, lapatinib monotherapy has limited effect on BCBM. Compared with the treatment of lapatinib alone, lapatinib plus capecitabine can significantly increase brain disease response rates in BCBM.^79^, ^80^, ^81^ Metro et al. reported the median brain‐specific PFS was 5.6 months in HER2+ BCBM patients treated with lapatinib plus capecitabine.^81^ In addition, in the LANDSCAPE study, lapatinib plus capecitabine was investigated as a first‐line treatment among patients with untreated brain metastases, with a CNS ORR of 57·1%.^82^

Neratinib is an irreversible pan‐HER TKI that inhibits HER1, HER2, and HER4. A phase II trial (TBCRC 022) suggested that neratinib plus capecitabine were active in refractory HER2+ BCBM. CNS ORR was 33% in the lapatinib‐treated cohort and 49% in the lapatinib –naïve cohort.^83^ Subsequent phase III trial (NALA) compared the efficacy between neratinib plus capecitabine and lapatinib plus capecitabine in patients with HER2‐positive advanced breast cancer, who had previously received at least 2 HER2‐targeted therapy regimens. The CNS ORR of lapatinib was lower than that of neratinib (15% vs. 26%).^84^, ^85^

Pyrotinib, a novel irreversible pan‐ERBB inhibitor (HER1, HER2, and HER4), has demonstrated its potent tumor‐suppressing activity in previous clinical trials.^86^, ^87^ The recent PERMEATE study reported promising activity of pyrotinib in combination with capecitabine against BCBM, with a CNS ORR of 74·6% of pyrotinib without previous radiotherapy and 42·1% of pyrotinib with previous radiotherapy. Furthermore, consistent activity was observed in extracranial metastatic lesions.^88^ In contrast, several studies proved the efficiency of pyrotinib plus radiotherapy.^89^, ^90^ The first real‐world study by Lin et al. using pyrotinib to treat HER2‐positive patients with BCBM showed that the pyrotinib‐based regimen plus radiotherapy had better intracranial control (ORR 66.7%) compared with the patients who did not have radiotherapy (ORR 6.3%).^90^ Tian et al. used pyrotinib in combination with radiotherapy and capecitabine in HER2‐positive BCBM patients, which discovered that pyrotinib could substantially increase the radiosensitivity. Moreover, they identified this finding by culturing HER2+ breast cancer cell lines in vitro.^89^ Whether pyrotinib plus radiotherapy can exactly enhance the treatment efficiency of BCBM patients requires further verification. Taken together, the development of TKIs has made a significant contribution to the therapy of breast cancer.

### 3.3 Immunotherapy

Despite the fact that immunotherapy has shown potent anti‐tumor activity in a variety of cancers, its application in breast cancer remains limited, and it shows promising activity only in metastatic TNBC. The clinical trial IMpassion130 investigated the effect of an immune checkpoint inhibitor atezolizumab plus nab‐paclitaxel in metastatic TNBC, suggesting that the combination prolonged PFS. However, the subgroup analysis did not show a benefit for patients with BCBM.^91^ However, we have to analyze these results prudently as the study population of CNS metastasis is very small, accounting for only 6.3%. A Phase II study is being investigated to assess the effectiveness and safety of treatment options for BCBM based on molecular subtype. Patients are divided into two cohorts by HR status and HER2 status. HER2+/HR‐ cohort receive pyrotinib plus temozolomide, and HER2‐/HR‐cohort receive bevacizumab, SHR1316 (a new anti‐PD‐L1 antibody), and cisplatin/carboplatin (NCT04303988). Additionally, new therapeutic strategies are being explored. For example, HER2‐CART cells were delivered into the brain's ventricles, which may recognize and kill tumor cells. Phase I trial is ongoing to evaluate the side effects and effectiveness of HER2‐CART cells in HER2‐positive BCBM (NCT03696030). Immune checkpoint inhibitors in combination with radiotherapy are presently being evaluated as well. The ongoing phase I/II trial is to evaluate the role of pembrolizumab and stereotactic radiosurgery (SRS) in BCBM patients (NCT03449238). A phase II clinical trial is studying the combination of atezolizumab and SRS as a possible treatment for TNBC with CNS metastasis (NCT03483012).

### 3.4 CDK4/6 inhibitors

Cyclin‐dependent kinases (CDKs) control the transition from one stage of the cell cycle to the next, and CDKs are activated upon interaction with their partner cyclins. CDK4 and CDK6, a pair of kinases that are similar to each other in structure and function, mediate transition from G0/G1‐phase to S‐phase of the cell cycle.^92^ The CDK 4/6 inhibitors such as palbociclib, ribociclib, abemaciclib, and dalpiciclib are a new class of drugs that interrupt the proliferation of cancer cells by inhibiting cell cycle progression. Previous studies have demonstrated the robust antitumor activity of CDK 4/6 inhibitors in HR+/HER2‐ breast cancer.^93^, ^94^, ^95^, ^96^ However, few studies have included BCBM patients. Abemaciclib is a selective CDK 4/6 inhibitor. Previous studies have shown that abemaciclib and its metabolites are more likely to cross the BBB than palbociclib, ribociclib, and dalpiciclib. Investigators applied abemaciclib to human xenograft models, which suggested that tumor growth decreased in the brain, and abemaciclib had the highest unbound brain‐to‐plasma ratio, displaying effective penetration to the brain.^97^ A phase II study of abemaciclib evaluated intracranial ORR of applying abemaciclib to patients with brain metastases secondary to HR‐positive breast cancer, suggesting an intracranial clinical benefit rate of 24% in heavily pretreated HR+ HER2‐ BCBM. However, this study did not meet its primary endpoint, with an intracranial ORR of 5.2%.^98^ The efficacy of palbociclib, ribociclib, and dalpiciclib in treating brain metastases is an important unanswered problem in the clinic. Prospective trials are being underway to investigate brain penetration and efficacy of abemaciclib (NCT02308020) and palbociclib (NCT02896335 and NCT04334330) in the treatment of BCBM.

### 3.5 Poly(adenosine diphosphate–ribose) polymerase (PARP) inhibitors

BRCA1 and BRCA2 are both associated with homologous recombination‐mediated DNA repair, checkpoint control of cell cycle, and transcription.^99^ The tumor suppressor BRCA is a part of the complex responsible for double‐stranded DNA breakages. Patients carrying BRCA1 and/or BRCA2 mutations lack the function of homologous recombinational repair of the single‐strand breaks, thus, remarkably increasing the risks of ovarian cancer and breast cancer. Several studies have highlighted that breast cancer patients carrying germline BRCA1 and/or BRCA2 mutations are more likely to have CNS metastasis.^100^, ^101^ PARP inhibitors target BRCA mutation and induce cell apoptosis by inhibiting the enzyme PARP from repairing single‐strand breaks.

PARP inhibitors (olaparib and talazoparib) have been approved by FDA for germline BRCA‐mutated breast cancer. The sEMBRACA trial was performed to evaluate patients with BRCA‐mutated metastatic breast cancer. In the talazoparib treatment group, 14.6% of patients had pretreatment and stable CNS lesions at baseline. Moreover, according to the subgroup analysis, the PFS benefit for patients with brain metastasis was superior to that for patients without brain metastasis, which implied that talazoparib may have an effect on CNS.^102^ In addition, the OlympiAD trial was done to investigate the role of olaparib in advanced breast cancer with BRCA mutation. It was found that although there was no statistical significance in the improvement of OS with olaparib compared to the treatment of physician's choice (TPC), the PFS of the olaparib group was longer than that of TPC (7.0 vs. 4.2 months). However, the effect on patients with brain metastasis was not reported in the subset analysis. The role of olaparib in BCBM warrants further studies.^103^, ^104^ Veliparib is regarded as an effective oral PARP inhibitor and presents antitumor activity in metastatic brain lesions. A phase I clinical trial has confirmed the effect and safety of veliparib plus whole‐brain radiotherapy (WBRT) for patients with CNS metastasis.^105^ The phase II trial has been carried out to compare veliparib plus WBRT with placebo plus WBRT in patients with brain metastases from non‐small cell lung cancer, which is still in process (NCT01657799). The phase III BROCADE3 trial, involving 5% of BCBM patients, compared veliparib versus placebo plus carboplatin‐paclitaxel in patients with HER2 negative metastatic breast cancer with a germline BRCA1 or BRCA2 mutation. Compared with placebo plus carboplatin‐paclitaxel, the PFS of the combination of veliparib and carboplatin‐paclitaxel was improved (14.5 vs. 12.6 months). While the subgroup analysis showed that no improvement of PFS was observed in veliparib plus carboplatin‐paclitaxel compared to carboplatin‐paclitaxel for germline BRCA mutation advanced breast cancer (8.3 vs. 12.5 months). Importantly, patients with brain metastasis should be interpreted with caution due to the small study population.^106^

## 4 FUTURE DIRECTIONS

Although several drugs have confirmed favorable outcomes in clinical trials, it is still imperative to detect brain metastases early and develop efficacious regimens for patients with progressive brain metastases as drug resistance is inevitable. In addition, it has been proven that metastatic cancers acquire genomic alterations during disease progression. Intracranial diseases have a distinctive genomic landscape different from the primary tumor and extracranial metastatic lesions.^25^ Thus, it is necessary to monitor the genetic variations of intracranial lesions to better implement individualized treatment. However, it is challenging to obtain intracranial lesions for gene detection from BCBM patients because of the complexity of neurosurgery and inherent risks. Circulating tumor DNA (ctDNA) released by tumor cells is a minimally liquid biopsy, which is used to monitor tumor progression, identify tumor genomic alterations, and track patients' response to treatment. However, plasma ctDNA may not accurately reflect the tumor burden of CNS.^107^ Generally, CSF is closely linked to CNS cancers, and CSF ctDNA is more abundantly present than plasma ctDNA in CNS cancers.^108^ ctDNA biomarkers provide real‐time assessment of tumor dynamics and play an essential role in selecting the best therapy and monitoring treatment efficacy.^109^

As previously mentioned, an intact BBB may hinder trastuzumab penetration to the brain. To improve the permeability of drugs to the CNS, two methods are being studied to overcome the BBB: (a) disrupting the BBB, such as intrathecal administration of the antibodies, intra‐arterial administration, radiotherapy to increase the BBB permeability and osmotherapy; (b) methods without disrupting the BBB, for example, increasing the dose of drugs or in combination with other therapeutic agents, nano‐functionalization of drugs to cross the BBB or intranasal administration.

Intrathecal administration helps drugs to enter the CNS via the lymphatic system.^110^ The molecular weight, drugs' biochemical features, and the BBB's efflux systems determine whether drugs can diffuse into deeper brain areas.^110^ It has been reported that direct intrathecal injections of trastuzumab can treat meningeal carcinomatosis resulted from breast cancer.^111^ In addition, a clinical trial has been implemented to determine the antitumor activity of intrathecal trastuzumab administration in advanced breast cancer patients with carcinomatous meningitis, which is still ongoing (NCT01373710). Osmotherapy produces a temporary, reversible disruption of BBB by causing endothelial cell shrinkage and thus opening the tight junctions. Osmotherapy is complicated, including an intra‐arterial infusion of mannitol (25%) into a carotid or vertebral artery, followed by intra‐arterial delivery of chemotherapy to treat brain tumors.^112^ Although osmotherapy is safe, it is hard to implement in clinical practice as it requires hospitalization with intra‐arterial cranial catheter placement under general anesthesia.^113^ Based on the latest PERMEATE study, there is a better CNS ORR in pyrotinib without previous radiotherapy than in pyrotinib with prior radiotherapy.^88^ Nevertheless, a number of studies hold that radiotherapy can increase the permeability of BBB.^89^, ^90^ Therefore, whether radiotherapy can improve the penetrability of BBB still needs further research.

Whether it is helpful to increase the prescription dose may warrant further study. A phase II study was carried out to identify the efficacy of pertuzumab plus high‐dose trastuzumab in BCBM patients and found a modest clinical benefit with a CNS ORR of 11%.^63^ A combination treatment strategy has become a cornerstone in treating terminal tumors as it enhances the anti‐tumor effect and conquers drug resistance to a certain degree. NEO100 is a high‐purity version of the natural monoterpene perillyl alcohol. Wang et al. detected that NEO100 could open the BBB reversibly and safely in mouse models, thus enabling brain entry of various‐sized therapeutics effectively.^114^ Subsequent research by the same team further displayed that intra‐arterial administration of NEO100 increased the ability of trastuzumab and T‐DM1 to penetrate BBB in vitro and in vivo and access to intracranial tumor lesions, thus providing a striking therapeutic activity. What's more, they discovered that the opening of BBB by NEO100 increased the recruitment of macrophages, mature NK cells, and CD8+ T cells to the tumor microenvironment.^115^ Furthermore, nanotherapy is an emerging technology. In a few preclinical studies, the role of nanotherapy in brain metastasis was investigated by using nanoparticles carrying anticancer agents to deliver drugs.^116^, ^117^, ^118^ Unfortunately, the number of clinical trials concerning nanotherapy in BCBM is too small, and there are no clinical data to support the idea that nanotherapy is superior to current treatment strategies. Thus, the application of nanotherapy is still controversial.

## 5 CONCLUSIONS

The treatment of advanced breast cancer has made important progress in the past 20 years, while, CNS metastasis remains the primary concern. CNS metastasis is identified as the leading cause of mortality in breast cancer patients. Local therapy, including surgery and radiotherapy, remains the standard treatment currently. However, cognitive impairment is inevitable even though surgery and radiotherapy have improved the survival of metastatic brain tumors. Especially, the WBRT significantly decreases the quality of life for patients. Then, systemic therapy plays an increasingly important role in the treatment of BCBM. Some targeted therapies focusing on underlying molecular changes and signaling pathways have presented potent antitumor activity against metastatic brain tumors, such as tucatinib, neratinib, and pyrotinib. Besides, the molecular characteristics of CNS metastasis differ from those of the primary tumors and metastases to other sites, reflecting the inherent heterogeneity of tumor. Hence, it is necessary to realize individual treatment through a comprehensive understanding of the gene changes of CNS metastasis. Additionally, CSF ctDNA can provide real‐time tumor dynamics assessment and plays a vital role in selecting the best therapy.

BBB can maintain homeostasis in the internal environment, but it may prevent some drugs like trastuzumab from penetrating the brain. Thus, the majority of drugs have a limited effect on brain metastases. At present, researchers are constantly trying to explore multiple methods to improve the permeability of the CNS. However, intracranial lesions are more likely to develop rapid resistance to systemic therapy. Therefore, more effective therapies for CNS metastasis are strongly needed. We believe that in the near future, new targeted therapies, immunotherapy, or multi‐modality of treatment can further improve the survival of BCBM patients.

## AUTHOR CONTRIBUTION

Hongna Sun: Conceptualization; Data curation; investigation; writing – original draft; writing – review & editing. Junnan Xu: Conceptualization; Data curation; investigation; writing – original draft; writing – review & editing. Shuang Dai: Conceptualization; investigation; writing – original draft; writing – review & editing. Yiwen Ma: Conceptualization; investigation; writing – original draft; writing – review & editing. Tao Sun: Conceptualization; Data curation; Investigation; Project administration; Writing – original draft; Writing – review & editing.

## FUNDING INFORMATION

This study was partly funded by Liaoning Province Key Laboratory Project of Breast Cancer Research (2016‐26‐1, ST), Shenyang Breast Cancer Clinical Medical Research Center (2020‐48‐3‐1, ST), Medical‐Engineering Cross Research Fund between Liaoning Cancer Hospital & Dalian University of Technology (LD202022, ST), “Metabolic Abnormality and Tumor” Research Project (ZP202017, ST), Beijing Medical Award Foundation (YXJL‐2020‐0941‐0752, ST), Wu Jieping Medical Foundation (320.6750.2020‐12‐21, 320.6750.2020‐6‐30, 320.6750.18541, ST).

## CONFLICT OF INTEREST

None.

## ETHICS STATEMENT

Neither informed consent to participate nor ethical approval is required.

## Data Availability

This review was based on published literature, all of which is fully listed.

## References

[1] Siegel RL , Miller KD , Jemal A . Cancer statistics, 2020. CA Cancer J Clin. 2020;70:7‐30. 31912902 10.3322/caac.21590

[2] Darlix A , Louvel G , Fraisse J , et al. Impact of breast cancer molecular subtypes on the incidence, kinetics and prognosis of central nervous system metastases in a large multicentre real‐life cohort. Br J Cancer. 2019;121:991‐1000. 31719684 10.1038/s41416-019-0619-y PMC6964671

[3] Sperduto PW , Berkey B , Gaspar LE , Mehta M . Curran W A new prognostic index and comparison to three other indices for patients with brain metastases: an analysis of 1,960 patients in the RTOG database. Int J Radiat Oncol Biol Phys. 2008;70:510‐514. 17931798 10.1016/j.ijrobp.2007.06.074

[4] Sperduto PW , Kased N , Roberge D , et al. Effect of tumor subtype on survival and the graded prognostic assessment for patients with breast cancer and brain metastases. Int J Radiat Oncol Biol Phys. 2012;82:2111‐2117. 21497451 10.1016/j.ijrobp.2011.02.027 PMC3172400

[5] Ehrlich P. Das Sauerstoff‐Bedürfniss des Organismus: eine farbenanalytische Studie. A Hirschwald,1885.

[6] Segarra M , Aburto MR , Acker‐Palmer A . Blood‐brain barrier dynamics to maintain brain homeostasis. Trends Neurosci. 2021;44:393‐405. 33423792 10.1016/j.tins.2020.12.002

[7] Langen UH , Ayloo S , Gu C . Development and cell biology of the blood‐brain barrier. Annu Rev Cell Dev Biol. 2019;35:591‐613. 31299172 10.1146/annurev-cellbio-100617-062608 PMC8934576

[8] Terstappen GC , Meyer AH , Bell RD , Zhang W . Strategies for delivering therapeutics across the blood‐brain barrier. Nat Rev Drug Discov. 2021;20:362‐383. 33649582 10.1038/s41573-021-00139-y

[9] Hajal C , Le Roi B , Kamm RD , Maoz BM . Biology and models of the blood‐brain barrier. Annu Rev Biomed Eng. 2021;23:359‐384. 34255993 10.1146/annurev-bioeng-082120-042814

[10] Eichler AF , Chung E , Kodack DP , Loeffler JS , Fukumura D , Jain RK . The biology of brain metastases‐translation to new therapies. Nat Rev Clin Oncol. 2011;8:344‐356. 21487419 10.1038/nrclinonc.2011.58 PMC3259742

[11] Arvanitis CD , Ferraro GB , Jain RK . The blood‐brain barrier and blood‐tumour barrier in brain tumours and metastases. Nat Rev Cancer. 2020;20:26‐41. 31601988 10.1038/s41568-019-0205-x PMC8246629

[12] De Craene B , Berx G . Regulatory networks defining EMT during cancer initiation and progression. Nat Rev Cancer. 2013;13:97‐110. 23344542 10.1038/nrc3447

[13] Liu SJ , Dang HX , Lim DA , Feng FY , Maher CA . Long noncoding RNAs in cancer metastasis. Nat Rev Cancer. 2021;21:446‐460. 33953369 10.1038/s41568-021-00353-1 PMC8288800

[14] Klemm F , Bleckmann A , Siam L , et al. β‐catenin‐independent WNT signaling in basal‐like breast cancer and brain metastasis. Carcinogenesis. 2011;32:434‐442. 21173432 10.1093/carcin/bgq269

[15] Smid M , Wang Y , Zhang Y , et al. Subtypes of breast cancer show preferential site of relapse. Cancer Res. 2008;68:3108‐3114. 18451135 10.1158/0008-5472.CAN-07-5644

[16] Nam D‐H , Jeon H‐M , Kim S , et al. Activation of notch signaling in a xenograft model of brain metastasis. Clin Cancer Res. 2008;14:4059‐4066. 18593982 10.1158/1078-0432.CCR-07-4039

[17] Xing F , Kobayashi A , Okuda H , et al. Reactive astrocytes promote the metastatic growth of breast cancer stem‐like cells by activating Notch signalling in brain. EMBO Mol Med. 2013;5:384‐396. 23495140 10.1002/emmm.201201623 PMC3598079

[18] Leontovich AA , Jalalirad M , Salisbury JL , et al. NOTCH3 expression is linked to breast cancer seeding and distant metastasis. Breast Cancer Res BCR. 2018;20:105. 30180881 10.1186/s13058-018-1020-0 PMC6123953

[19] Dey N , De P , Leyland‐Jones B . PI3K‐AKT‐mTOR inhibitors in breast cancers: from tumor cell signaling to clinical trials. Pharmacol Ther. 2017;175:91‐106. 28216025 10.1016/j.pharmthera.2017.02.037

[20] Schmit F , Utermark T , Zhang S , et al. PI3K isoform dependence of PTEN‐deficient tumors can be altered by the genetic context. Proc Natl Acad Sci USA. 2014;111:6395‐6400. 24737887 10.1073/pnas.1323004111 PMC4035990

[21] Braccini L , Ciraolo E , Campa CC , et al. PI3K‐C2γ is a Rab5 effector selectively controlling endosomal Akt2 activation downstream of insulin signalling. Nat Commun. 2015;6:7400. 26100075 10.1038/ncomms8400 PMC4479417

[22] Miricescu D , Totan A , Stanescu‐Spinu I‐I , et al. PI3K/AKT/mTOR signaling pathway in breast cancer: from molecular landscape to clinical aspects. Int J Mol Sci. 2020;22:173. 33375317 10.3390/ijms22010173 PMC7796017

[23] Lim W , Mayer B , Pawson T . Cell Signaling. Taylor & Francis; 2014.

[24] Tehranian C , Fankhauser L , Harter PN , et al. The PI3K/Akt/mTOR pathway as a preventive target in melanoma brain metastasis. Neuro Oncol. 2022;24:213‐225. 34216217 10.1093/neuonc/noab159 PMC8804893

[25] Morgan AJ , Giannoudis A , Palmieri C . The genomic landscape of breast cancer brain metastases: a systematic review. Lancet Oncol. 2021;22:e7‐e17. 33387511 10.1016/S1470-2045(20)30556-8

[26] Blazquez R , Wlochowitz D , Wolff A , et al. PI3K: a master regulator of brain metastasis‐promoting macrophages/microglia. Glia. 2018;66:2438‐2455. 30357946 10.1002/glia.23485

[27] Lee JJ , Loh K . Yap Y‐S PI3K/Akt/mTOR inhibitors in breast cancer. Cancer Biol Med. 2015;12:342‐354. 26779371 10.7497/j.issn.2095-3941.2015.0089 PMC4706528

[28] Hohensee I , Lamszus K , Riethdorf S , et al. Frequent genetic alterations in EGFR‐ and HER2‐driven pathways in breast cancer brain metastases. Am J Pathol. 2013;183:83‐95. 23665199 10.1016/j.ajpath.2013.03.023

[29] Adamo B , Deal AM , Burrows E , et al. Phosphatidylinositol 3‐kinase pathway activation in breast cancer brain metastases. Breast Cancer Res BCR. 2011;13:R125. 22132754 10.1186/bcr3071 PMC3326567

[30] Wikman H , Lamszus K , Detels N , et al. Relevance of PTEN loss in brain metastasis formation in breast cancer patients. Breast Cancer Research BCR. 2012;14:R49. 22429330 10.1186/bcr3150 PMC3446383

[31] Zhang L , Zhang S , Yao J , et al. Microenvironment‐induced PTEN loss by exosomal microRNA primes brain metastasis outgrowth. Nature. 2015;527:100‐104. 26479035 10.1038/nature15376 PMC4819404

[32] Hsu JL , Hung M‐C . The role of HER2, EGFR, and other receptor tyrosine kinases in breast cancer. Cancer Metastasis Rev. 2016;35:575‐588. 27913999 10.1007/s10555-016-9649-6 PMC5215954

[33] Yarden Y , Sliwkowski MX . Untangling the ErbB signalling network. Nat Rev Mol Cell Biol. 2001;2:127‐137. 11252954 10.1038/35052073

[34] Baselga J , Swain SM . Novel anticancer targets: revisiting ERBB2 and discovering ERBB3. Nat Rev Cancer. 2009;9:463‐475. 19536107 10.1038/nrc2656

[35] Bhargava R , Gerald WL , Li AR , et al. EGFR gene amplification in breast cancer: correlation with epidermal growth factor receptor mRNA and protein expression and HER‐2 status and absence of EGFR‐activating mutations. Modern Pathol. 2005;18:1027‐1033. 10.1038/modpathol.3800438 15920544

[36] Kodack DP , Askoxylakis V , Ferraro GB , et al. The brain microenvironment mediates resistance in luminal breast cancer to PI3K inhibition through HER3 activation. Sci Transl Med. 2017;9:eaal4682. 28539475 10.1126/scitranslmed.aal4682 PMC5917603

[37] Koganemaru S , Kuboki Y , Koga Y , et al. U3‐1402, a novel HER3‐targeting antibody‐drug conjugate, for the treatment of colorectal cancer. Mol Cancer Ther. 2019;18:2043‐2050. 31395690 10.1158/1535-7163.MCT-19-0452

[38] Gil V , Miranda S , Riisnaes R , et al. HER3 is an actionable target in advanced prostate cancer. Cancer Res. 2021;81:6207‐6218. 34753775 10.1158/0008-5472.CAN-21-3360 PMC8932336

[39] Yonesaka K . HER2‐/HER3‐targeting antibody‐drug conjugates for treating lung and colorectal cancers resistant to EGFR inhibitors. Cancer. 2021;13:1047. 10.3390/cancers13051047 PMC7958627 33801379

[40] Jänne PA , Baik C , Su W‐C , et al. Efficacy and safety of patritumab deruxtecan (HER3‐DXd) in EGFR inhibitor‐resistant, −mutated non‐small cell lung cancer. Cancer Discov. 2022;12:74‐89. 34548309 10.1158/2159-8290.CD-21-0715 PMC9401524

[41] Krop IE , Masuda N , Mukohara T , et al. Results from the phase 1/2 study of patritumab deruxtecan, a HER3‐directed antibody‐drug conjugate (ADC), in patients with HER3‐expressing metastatic breast cancer (MBC). J Clin Oncol. 2022;40:1002. 10.1200/JCO.23.00882 PMC10730028 37801674

[42] Zuo T , Wang L , Morrison C , et al. FOXP3 is an X‐linked breast cancer suppressor gene and an important repressor of the HER‐2/ErbB2 oncogene. Cell. 2021;184:6378. 34942100 10.1016/j.cell.2021.11.030

[43] Moasser MM . Targeting the function of the HER2 oncogene in human cancer therapeutics. Oncogene. 2007;26:6577‐6592. 17486079 10.1038/sj.onc.1210478 PMC3071580

[44] Aversa C , Rossi V , Geuna E , et al. Metastatic breast cancer subtypes and central nervous system metastases. Breast (Edinburgh, Scotland). 2014;23:623‐628. 24993072 10.1016/j.breast.2014.06.009

[45] Niikura N , Hayashi N , Masuda N , et al. Treatment outcomes and prognostic factors for patients with brain metastases from breast cancer of each subtype: a multicenter retrospective analysis. Breast Cancer Res Treat. 2014;147:103‐112. 25106661 10.1007/s10549-014-3090-8

[46] Brosnan EM , Anders CK . Understanding patterns of brain metastasis in breast cancer and designing rational therapeutic strategies. Ann Transl Med. 2018;6:163. 29911111 10.21037/atm.2018.04.35 PMC5985267

[47] Palmieri D , Bronder JL , Herring JM , et al. Her‐2 overexpression increases the metastatic outgrowth of breast cancer cells in the brain. Cancer Res. 2007;67:4190‐4198. 17483330 10.1158/0008-5472.CAN-06-3316

[48] Brastianos PK , Carter SL , Santagata S , et al. Genomic characterization of brain metastases reveals branched evolution and potential therapeutic targets. Cancer Discov. 2015;5:1164‐1177. 26410082 10.1158/2159-8290.CD-15-0369 PMC4916970

[49] Saunus JM , Quinn MCJ , Patch A‐M , et al. Integrated genomic and transcriptomic analysis of human brain metastases identifies alterations of potential clinical significance. J Pathol. 2015;237:363‐378. 26172396 10.1002/path.4583

[50] Baselga J , Im S‐A , Iwata H , et al. Buparlisib plus fulvestrant versus placebo plus fulvestrant in postmenopausal, hormone receptor‐positive, HER2‐negative, advanced breast cancer (BELLE‐2): a randomised, double‐blind, placebo‐controlled, phase 3 trial. Lancet Oncol. 2017;18:904‐916. 28576675 10.1016/S1470-2045(17)30376-5 PMC5549667

[51] Di Leo A , Johnston S , Lee KS , et al. Buparlisib plus fulvestrant in postmenopausal women with hormone‐receptor‐positive, HER2‐negative, advanced breast cancer progressing on or after mTOR inhibition (BELLE‐3): a randomised, double‐blind, placebo‐controlled, phase 3 trial. Lancet Oncol. 2018;19:904‐916. 29223745 10.1016/S1470-2045(17)30688-5

[52] Ippen FM , Grosch JK , Subramanian M , et al. Targeting the PI3K/Akt/mTOR pathway with the pan‐Akt inhibitor GDC‐0068 in PIK3CA‐mutant breast cancer brain metastases. Neuro Oncol. 2019;21:1401‐1411. 31173106 10.1093/neuonc/noz105 PMC6827829

[53] Baselga J , Campone M , Piccart M , et al. Everolimus in postmenopausal hormone‐receptor‐positive advanced breast cancer. N Engl J Med. 2012;366:520‐529. 22149876 10.1056/NEJMoa1109653 PMC5705195

[54] André F , O'Regan R , Ozguroglu M , et al. Everolimus for women with trastuzumab‐resistant, HER2‐positive, advanced breast cancer (BOLERO‐3): a randomised, double‐blind, placebo‐controlled phase 3 trial. Lancet Oncol. 2014;15:580‐591. 24742739 10.1016/S1470-2045(14)70138-X

[55] Hurvitz S , Singh R , Adams B , et al. Phase Ib/II single‐arm trial evaluating the combination of everolimus, lapatinib and capecitabine for the treatment of HER2‐positive breast cancer with brain metastases (TRIO‐US B‐09). Therap Adv Med Oncol. 2018;10:1758835918807339. 30542377 10.1177/1758835918807339 PMC6236634

[56] Van Swearingen AED , Siegel MB , Deal AM , et al. LCCC 1025: a phase II study of everolimus, trastuzumab, and vinorelbine to treat progressive HER2‐positive breast cancer brain metastases. Breast Cancer Res Treat. 2018;171:637‐648. 29938395 10.1007/s10549-018-4852-5 PMC6779035

[57] Vogel CL , Cobleigh MA , Tripathy D , et al. Efficacy and safety of trastuzumab as a single agent in first‐line treatment of HER2‐overexpressing metastatic breast cancer. J Clin Oncol. 2002;20:719‐726. 11821453 10.1200/JCO.2002.20.3.719

[58] Brufsky AM , Mayer M , Rugo HS , et al. Central nervous system metastases in patients with HER2‐positive metastatic breast cancer: incidence, treatment, and survival in patients from registHER. Clin Cancer Res. 2011;17:4834‐4843. 21768129 10.1158/1078-0432.CCR-10-2962

[59] Dawood S , Broglio K , Esteva FJ , et al. Defining prognosis for women with breast cancer and CNS metastases by HER2 status. Ann Oncol. 2008;19:1242‐1248. 18334512 10.1093/annonc/mdn036

[60] Olson EM , Abdel‐Rasoul M , Maly J , Wu CS , Lin NU , Shapiro CL . Incidence and risk of central nervous system metastases as site of first recurrence in patients with HER2‐positive breast cancer treated with adjuvant trastuzumab. Ann Oncol. 2013;24:1526‐1533. 23463626 10.1093/annonc/mdt036 PMC3660080

[61] Swain SM , Baselga J , Kim S‐B , et al. Pertuzumab, trastuzumab, and docetaxel in HER2‐positive metastatic breast cancer. N Engl J Med. 2015;372:724‐734. 25693012 10.1056/NEJMoa1413513 PMC5584549

[62] Swain SM , Baselga J , Miles D , et al. Incidence of central nervous system metastases in patients with HER2‐positive metastatic breast cancer treated with pertuzumab, trastuzumab, and docetaxel: results from the randomized phase III study CLEOPATRA. Ann Oncol. 2014;25:1116‐1121. 24685829 10.1093/annonc/mdu133 PMC4037862

[63] Lin NU , Pegram M , Sahebjam S , et al. Pertuzumab plus high‐dose trastuzumab in patients with progressive brain metastases and HER2‐positive metastatic breast cancer: primary analysis of a phase II study. J Clin Oncol. 2021;39:2667‐2675. 33945296 10.1200/JCO.20.02822 PMC8376355

[64] Krop IE , Lin NU , Blackwell K , et al. Trastuzumab emtansine (T‐DM1) versus lapatinib plus capecitabine in patients with HER2‐positive metastatic breast cancer and central nervous system metastases: a retrospective, exploratory analysis in EMILIA. Ann Oncol. 2015;26:113‐119. 25355722 10.1093/annonc/mdu486 PMC4679405

[65] Montemurro F , Delaloge S , Barrios CH , et al. Trastuzumab emtansine (T‐DM1) in patients with HER2‐positive metastatic breast cancer and brain metastases: exploratory final analysis of cohort 1 from KAMILLA, a single‐arm phase IIIb clinical trial. Ann Oncol. 2020;31:1350‐1358. 32634611 10.1016/j.annonc.2020.06.020

[66] Modi S , Saura C , Yamashita T , et al. Trastuzumab deruxtecan in previously treated HER2‐positive breast cancer. N Engl J Med. 2020;382:610‐621. 31825192 10.1056/NEJMoa1914510 PMC7458671

[67] Jerusalem GHM , Park YH , Yamashita T , et al. Trastuzumab deruxtecan (T‐DXd) in patients with HER2+ metastatic breast cancer with brain metastases: a subgroup analysis of the DESTINY‐Breast01 trial. J Clin Oncol. 2021;39:526.

[68] Cortés J , Kim S‐B , Chung W‐P , et al. Trastuzumab deruxtecan versus trastuzumab emtansine for breast cancer. N Engl J Med. 2022;386:1143‐1154. 35320644 10.1056/NEJMoa2115022

[69] Hamilton EP , Bragaia VPH , Yeo W , et al. Trastuzumab deruxtecan (T‐DXd) versus trastuzumab emtansine (T‐DM1) in patients (pts) with HER2‐positive (HER2+) unresectable and/or metastatic breast cancer (mBC): Safety follow‐up of the randomized, phase 3 study DESTINY‐Breast03. J Clin Oncol. 2022;40:1000.

[70] Pérez‐García JM , Batista MV , Cortez P , et al. Trastuzumab deruxtecan in patients with central nervous system involvement from HER2‐positive breast cancer: the DEBBRAH trial. Neuro Oncol. 2022;noac144. 10.1093/neuonc/noac144 PMC9825345 35639825

[71] Bartsch R , Berghoff A , Furtner J , et al. 165MO trastuzumab‐deruxtecan (T‐DXd) in HER2‐positive breast cancer patients (pts) with active brain metastases: primary outcome analysis from the TUXEDO‐1 trial. Ann Oncol. 2022;33:S198. 10.1093/neuonc/noae123 PMC11630562 38963808

[72] Modi S , Jacot W , Yamashita T , et al. Trastuzumab deruxtecan (T‐DXd) versus treatment of physician's choice (TPC) in patients (pts) with HER2‐low unresectable and/or metastatic breast cancer (mBC): Results of DESTINY‐Breast04, a randomized, phase 3 study. Am Soc Clin Oncol. 2022;40:LBA3.

[73] Olson EM , Najita JS , Sohl J , et al. Clinical outcomes and treatment practice patterns of patients with HER2‐positive metastatic breast cancer in the post‐trastuzumab era. Breast (Edinburgh, Scotland). 2013;22:525‐531. 23352568 10.1016/j.breast.2012.12.006 PMC3713786

[74] Gori S , Rimondini S , De Angelis V , et al. Central nervous system metastases in HER‐2 positive metastatic breast cancer patients treated with trastuzumab: incidence, survival, and risk factors. Oncologist. 2007;12:766‐773. 17673608 10.1634/theoncologist.12-7-766

[75] Pestalozzi BC , Brignoli S . Trastuzumab in CSF. J Clin Oncol. 2000;18:2349‐2351. 10.1200/JCO.2000.18.11.2349 10829059

[76] Murthy RK , Loi S , Okines A , et al. Tucatinib, trastuzumab, and capecitabine for HER2‐positive metastatic breast cancer. N Engl J Med. 2020;382:597‐609. 31825569 10.1056/NEJMoa1914609

[77] Cordero A , Ramsey MD , Kanojia D , et al. Combination of tucatinib and neural stem cells secreting anti‐HER2 antibody prolongs survival of mice with metastatic brain cancer. Proc Natl Acad Sci USA. 2022;119:1‐11. 10.1073/pnas.2112491119 PMC8740706 34969858

[78] Morikawa A , Peereboom DM , Thorsheim HR , et al. Capecitabine and lapatinib uptake in surgically resected brain metastases from metastatic breast cancer patients: a prospective study. Neuro Oncol. 2015;17:289‐295. 25015089 10.1093/neuonc/nou141 PMC4288517

[79] Lin NU , Carey LA , Liu MC , et al. Phase II trial of lapatinib for brain metastases in patients with human epidermal growth factor receptor 2‐positive breast cancer. J Clin Oncol. 2008;26:1993‐1999. 18421051 10.1200/JCO.2007.12.3588 PMC4524351

[80] Lin NU , Diéras V , Paul D , et al. Multicenter phase II study of lapatinib in patients with brain metastases from HER2‐positive breast cancer. Clin Cancer Res. 2009;15:1452‐1459. 19228746 10.1158/1078-0432.CCR-08-1080

[81] Metro G , Foglietta J , Russillo M , et al. Clinical outcome of patients with brain metastases from HER2‐positive breast cancer treated with lapatinib and capecitabine. Ann Oncol. 2011;22:625‐630. 20724575 10.1093/annonc/mdq434

[82] Bachelot T , Romieu G , Campone M , et al. Lapatinib plus capecitabine in patients with previously untreated brain metastases from HER2‐positive metastatic breast cancer (LANDSCAPE): a single‐group phase 2 study. Lancet Oncol. 2013;14:64‐71. 23122784 10.1016/S1470-2045(12)70432-1

[83] Freedman RA , Gelman RS , Anders CK , et al. TBCRC 022: a phase II trial of neratinib and capecitabine for patients with human epidermal growth factor receptor 2‐positive breast cancer and brain metastases. J Clin Oncol. 2019;37:1081‐1089. 30860945 10.1200/JCO.18.01511 PMC6494354

[84] Saura C , Oliveira M , Feng Y‐H , et al. Neratinib plus capecitabine versus lapatinib plus capecitabine in HER2‐positive metastatic breast cancer previously treated with ≥ 2 HER2‐directed regimens: phase III NALA trial. J Clin Oncol. 2020;38:3138‐3149. 32678716 10.1200/JCO.20.00147 PMC7499616

[85] Hurvitz SA , Saura C , Oliveira M , et al. Efficacy of neratinib plus capecitabine in the subgroup of patients with central nervous system involvement from the NALA trial. Oncologist. 2021;26:e1327‐e1338. 34028126 10.1002/onco.13830 PMC8342591

[86] Xu B , Yan M , Ma F , et al. Pyrotinib plus capecitabine versus lapatinib plus capecitabine for the treatment of HER2‐positive metastatic breast cancer (PHOEBE): a multicentre, open‐label, randomised, controlled, phase 3 trial. Lancet Oncol. 2021;22:351‐360. 33581774 10.1016/S1470-2045(20)30702-6

[87] Yan M , Bian L , Hu X , et al. Pyrotinib plus capecitabine for human epidermal growth factor receptor 2‐positive metastatic breast cancer after trastuzumab and taxanes (PHENIX): a randomized, double‐blind, placebo‐controlled phase 3 study. Transl Breast Cancer Res. 2020;1:13.

[88] Yan M , Ouyang Q , Sun T , et al. Pyrotinib plus capecitabine for patients with human epidermal growth factor receptor 2‐positive breast cancer and brain metastases (PERMEATE): a multicentre, single‐arm, two‐cohort, phase 2 trial. Lancet Oncol. 2022;23:353‐361. 35085506 10.1016/S1470-2045(21)00716-6

[89] Tian W , Hao S , Wang L , et al. Pyrotinib treatment enhances the radiosensitivity in HER2‐positive brain metastatic breast cancer patients. Anticancer Drugs. 2022;33:e622‐e627. 34407046 10.1097/CAD.0000000000001199

[90] Lin Y , Lin M , Zhang J , et al. Real‐world data of pyrotinib‐based therapy in metastatic her2‐positive breast cancer: promising efficacy in lapatinib‐treated patients and in brain metastasis. Cancer Res Treatm. 2020;52:1059‐1066. 10.4143/crt.2019.633 PMC7577809 32340083

[91] Schmid P , Adams S , Rugo HS , et al. Atezolizumab and nab‐paclitaxel in advanced triple‐negative breast cancer. N Engl J Med. 2018;379:2108‐2121. 30345906 10.1056/NEJMoa1809615

[92] O'Leary B , Finn RS . Turner NC Treating cancer with selective CDK4/6 inhibitors. Nat Rev Clin Oncol. 2016;13:417‐430. 27030077 10.1038/nrclinonc.2016.26

[93] Xu B , Zhang Q , Zhang P , et al. Dalpiciclib or placebo plus fulvestrant in hormone receptor‐positive and HER2‐negative advanced breast cancer: a randomized, phase 3 trial. Nat Med. 2021;27:1904‐1909. 34737452 10.1038/s41591-021-01562-9

[94] Cristofanilli M , Turner NC , Bondarenko I , et al. Fulvestrant plus palbociclib versus fulvestrant plus placebo for treatment of hormone‐receptor‐positive, HER2‐negative metastatic breast cancer that progressed on previous endocrine therapy (PALOMA‐3): final analysis of the multicentre, double‐blind, phase 3 randomised controlled trial. Lancet Oncol. 2016;17:425‐439. 26947331 10.1016/S1470-2045(15)00613-0

[95] Sledge GW , Toi M , Neven P , et al. The effect of abemaciclib plus fulvestrant on overall survival in hormone receptor‐positive, ERBB2‐negative breast cancer that progressed on endocrine therapy‐MONARCH 2: a randomized clinical trial. JAMA Oncol. 2020;6:116‐124. 31563959 10.1001/jamaoncol.2019.4782 PMC6777264

[96] Slamon DJ , Neven P , Chia S , et al. Overall survival with ribociclib plus fulvestrant in advanced breast cancer. N Engl J Med. 2020;382:514‐524. 31826360 10.1056/NEJMoa1911149

[97] Raub TJ , Wishart GN , Kulanthaivel P , et al. Brain exposure of two selective dual CDK4 and CDK6 inhibitors and the antitumor activity of CDK4 and CDK6 inhibition in combination with temozolomide in an intracranial glioblastoma xenograft. Drug Metab Dispos. 2015;43:1360‐1371. 26149830 10.1124/dmd.114.062745

[98] Tolaney SM , Sahebjam S , Le Rhun E , et al. A phase II study of abemaciclib in patients with brain metastases secondary to hormone receptor‐positive breast cancer. Clin Cancer Res. 2020;26:5310‐5319. 32694159 10.1158/1078-0432.CCR-20-1764

[99] Venkitaraman AR . Cancer susceptibility and the functions of BRCA1 and BRCA2. Cell. 2002;108:171‐182. 11832208 10.1016/s0092-8674(02)00615-3

[100] Song Y , Barry WT , Seah DS , Tung NM , Garber JE , Lin NU . Patterns of recurrence and metastasis in BRCA1/BRCA2‐associated breast cancers. Cancer. 2020;126:271‐280. 31581314 10.1002/cncr.32540 PMC7003745

[101] Albiges L , André F , Balleyguier C , Gomez‐Abuin G , Chompret A , Delaloge S . Spectrum of breast cancer metastasis in BRCA1 mutation carriers: highly increased incidence of brain metastases. Ann Oncol. 2005;16:1846‐1847. 15972278 10.1093/annonc/mdi351

[102] Litton JK , Rugo HS , Ettl J , et al. Talazoparib in patients with advanced breast cancer and a germline BRCA mutation. N Engl J Med. 2018;379:753‐763. 30110579 10.1056/NEJMoa1802905 PMC10600918

[103] Robson ME , Tung N , Conte P , et al. OlympiAD final overall survival and tolerability results: Olaparib versus chemotherapy treatment of physician's choice in patients with a germline BRCA mutation and HER2‐negative metastatic breast cancer. Ann Oncol. 2019;30:558‐566. 30689707 10.1093/annonc/mdz012 PMC6503629

[104] Robson M , Im S‐A , Senkus E , et al. Olaparib for metastatic breast cancer in patients with a germline BRCA mutation. N Engl J Med. 2017;377:523‐533. 28578601 10.1056/NEJMoa1706450

[105] Mehta MP , Wang D , Wang F , et al. Veliparib in combination with whole brain radiation therapy in patients with brain metastases: results of a phase 1 study. J Neurooncol. 2015;122:409‐417. 25682091 10.1007/s11060-015-1733-1

[106] Diéras V , Han HS , Kaufman B , et al. Veliparib with carboplatin and paclitaxel in BRCA‐mutated advanced breast cancer (BROCADE3): a randomised, double‐blind, placebo‐controlled, phase 3 trial. Lancet Oncol. 2020;21:1269‐1282. 32861273 10.1016/S1470-2045(20)30447-2

[107] Bettegowda C , Sausen M , Leary RJ , et al. Detection of circulating tumor DNA in early‐ and late‐stage human malignancies. Sci Transl Med. 2014;6:224ra224. 10.1126/scitranslmed.3007094 PMC4017867 24553385

[108] De Mattos‐Arruda L , Mayor R , Ng CKY , et al. Cerebrospinal fluid‐derived circulating tumour DNA better represents the genomic alterations of brain tumours than plasma. Nat Commun. 2015;6:8839. 26554728 10.1038/ncomms9839 PMC5426516

[109] De Mattos‐Arruda L . Liquid biopsy for HER2‐positive breast cancer brain metastasis: the role of the cerebrospinal fluid. ESMO Open. 2017;2:e000270. 29067217 10.1136/esmoopen-2017-000270 PMC5640134

[110] Paris J , Angeli E , Bousquet G . The pharmacology of xenobiotics after intracerebro spinal fluid administration: implications for the treatment of brain tumors. Int J Mol Sci. 2021;22:1281. 33525427 10.3390/ijms22031281 PMC7865853

[111] Platini C , Long J , Walter S . Meningeal carcinomatosis from breast cancer treated with intrathecal trastuzumab. Lancet Oncol. 2006;7:778‐780. 16945774 10.1016/S1470-2045(06)70864-6

[112] Angeli E , Nguyen TT , Janin A , Bousquet G . How to make anticancer drugs cross the blood‐brain barrier to treat brain metastases. Int J Mol Sci. 2019;21:22. 31861465 10.3390/ijms21010022 PMC6981899

[113] Haluska M , Anthony ML . Osmotic blood‐brain barrier modification for the treatment of malignant brain tumors. Clin J Oncol Nurs. 2004;8:263‐267. 15208820 10.1188/04.CJON.263-267

[114] Wang W , Marín‐Ramos NI , He H , et al. NEO100 enables brain delivery of blood–brain barrier impermeable therapeutics. Neuro Oncol. 2021;23:63‐75. 32877532 10.1093/neuonc/noaa206 PMC7850137

[115] Wang W , He H , Marín‐Ramos NI , et al. Enhanced brain delivery and therapeutic activity of trastuzumab after blood‐brain barrier opening by NEO100 in mouse models of brain‐metastatic breast cancer. Neuro Oncol. 2021;23:1656‐1667. 33659980 10.1093/neuonc/noab041 PMC8485439

[116] Zhang L , Zhao D . Applications of nanoparticles for brain cancer imaging and therapy. J Biomed Nanotechnol. 2014;10:1713‐1731. 25992438 10.1166/jbn.2014.1896

[117] He C , Cai P , Li J , et al. Blood‐brain barrier‐penetrating amphiphilic polymer nanoparticles deliver docetaxel for the treatment of brain metastases of triple negative breast cancer. Journal of controlled release: official journal of the controlled release. Society. 2017;246:98‐109. 10.1016/j.jconrel.2016.12.019 28017889

[118] Patil R , Ljubimov AV , Gangalum PR , et al. MRI virtual biopsy and treatment of brain metastatic tumors with targeted nanobioconjugates: nanoclinic in the brain. ACS Nano. 2015;9:5594‐5608. 25906400 10.1021/acsnano.5b01872 PMC4768903

